# The millipedes collected by the Museum "La Specola" on Madagascar 1989/1991, with the description of three new species of giant pill-millipedes (Diplopoda, Sphaerotheriida, Arthrosphaeridae)

**DOI:** 10.3897/zookeys.930.47620

**Published:** 2020-04-28

**Authors:** Thomas Wesener, Pooja Avinipully Anilkumar

**Affiliations:** 1 Zoological Research Museum Alexander Koenig (ZFMK), Leibniz Institute for Animal Biodiversity, Adenauerallee 160, D-53113, Bonn, Germany Zoological Research Museum Alexander Koenig Bonn Germany

**Keywords:** Biodiversity, *COI*, introduced species, Madagascar, museum collection

## Abstract

A large collection of millipedes (Diplopoda) from Madagascar, belonging to the Museum “La Specola” in Florence, Italy were investigated. The collection includes three new species of the giant pill-millipede genus *Zoosphaerium* Pocock, 1895 which are described here as *Zoosphaerium mangabe* Wesener, **sp. nov.**, *Z. bartolozzii* Anilkumar & Wesener, **sp. nov.**, and *Z. taitii* Anilkumar & Wesener, **sp. nov.**, all belonging to the *Z. coquerelianum* species group. The latter two are currently only known from a single site. Other specimens belonging to eight orders (Polyxenida, Sphaerotheriida, Polyzoniida, Siphonophorida, Chordeumatida, Polydesmida, Spirobolida, and Spirostreptida) are listed. Three tropical tramp species, *Pseudospirobolellus avernus* (Butler, 1876), *Glyphiulus granulatus* Gervais, 1847, and *Chondromorpha xanthotricha* (Attems, 1898) are recorded for the first time from Madagascar. New locality data is provided for *Zoosphaerium neptunus* (Butler, 1872), *Z. villosum* Wesener & Sierwald, 2005, *Z. blandum* (de Saussure & Zehntner, 1897), *Sphaeromimus musicus* (de Saussure & Zehntner, 1897), *Rhinotus purpureus* (Pocock, 1894), *Hylekobolus andasibensis* Wesener, 2009, *Aphistogoniulus infernalis* Wesener, 2009, *Ostinobolus rufus* Wesener, 2009, *Ostinobolus subterraneus* Wesener, 2009, *Dactylobolus bivirgatus* (Karsch, 1881), and *Eumekius antimena* (de Saussure & Zehntner, 1901).

## Introduction

Madagascar, the fourth largest island lying 400 km east of Africa in the Indian Ocean, is one of the world’s biodiversity hot-spots, great for the studies of endemism, species richness, and island gigantism (Myers et al. 2000, Goodman and Benstead 2005, Wesener and VandenSpiegel 2009). Madagascar with India were the first landmasses to be separated from Gondwana approximately 170 million years ago, subsequently split from India around 90-85 million years ago (Ali and Aitchison 2008). The long isolation of Madagascar has given rise to an enormous level of endemism, resulting in 96% of plants, 86% of macro-invertebrates (Goodman and Benstead 2005), 51% of birds, 90% of mammals, 99% of amphibians, and more than 90% of reptiles (Harper et al. 2007) being endemic. Madagascar harbors various forest types, humid rainforests on the east coast (Harper et al. 2007), the montane forests at the center, tropical dry forests in the west, desert spiny forests in the southwest and tropical littoral forests on the eastern shore. Such an insularity and habitat diversity aided the micro-endemism and the speciation observable in different plant and animal taxa on Madagascar (Goodman 2007).

Soil fauna is a species-rich component of terrestrial ecosystems, where one of the major faunal elements is arthropods, especially terrestrial insects (Giller 1996). Flightless arthropods are more prone to speciation because of their light body weight, shorter generation time, and smaller size requirements of habitat compared to other animal groups (Brühl 1997). Millipedes (class Diplopoda) are major detritivores in all types of forests (Golovatch and Kime 2009) and one of the eye-catching macro-invertebrate group on Madagascar (Wesener 2009, Sagorny and Wesener 2017). The giant pill-millipedes (order Sphaerotheriida) are the most diverse myriapod group on Madagascar with 81 known strictly endemic species. They also show micro-endemism and island gigantism (Wesener and Wägele 2008, Wesener et al. 2010a, b). Among the Sphaerotheriida family Arthrosphaeridae, three of the four genera, *Zoosphaerium* Pocock, 1895, *Microsphaerotherium* Wesener & VandenSpiegel, 2007, and *Sphaeromimus* de Saussure & Zehntner, 1902 are endemic to Madagascar while the genus *Arthrosphaera* Pocock, 1895 occurs in southern India and Sri Lanka (Wesener and VandenSpiegel 2009, Golovatch and Wesener 2016).

Morphological and molecular studies show that the Malagasy genus *Sphaeromimus* is more closely related to the Indian genus *Arthrosphaera*, which reflects an Indian-Malagasy biogeographical affinity (Wesener and VandenSpiegel 2009, Wesener et al. 2010a, 2014, Moritz and Wesener 2017). Within the family Arthrosphaeridae, the endemic Malagasy genus *Zoosphaerium* has the highest number of known species (67) (Wesener 2016, Sagorny and Wesener 2017). Some species of *Zoosphaerium* show island gigantism; thus, the female of *Z. neptunus* (Butler, 1872), with a length of 80.9 mm and when rolled-up the size of a tennis ball, is the largest described species of all Sphaerotheriida (Wesener and Wägele 2008).

Deforestation is a key cause of species extinction on Madagascar (Harper et al. 2007). Madagascar has undergone an enormous amount of deforestation in the past years, resulting in only 9.9% of natural forests remaining (Myers et al. 2000). The arrival of humans on Madagascar dates back to 2000 years and has changed the land structure especially by forest fragmentation for agriculture and charcoal production (Burney 2003). During the past 50 years, approximately 40% of the remaining forests on Madagascar were deforested (Harper et al. 2007) and this destruction is still continuing today. The region prone to highest percentage of deforestation is the spiny forest, with a reduction of 28% in last two decades (Harper et al. 2007). Because of this massive deforestation, 65 species of *Zoosphaerium* are listed on the IUCN Red List (IUCN 2019), where seven are critically endangered, three are endangered, three are vulnerable, and 18 are nearly threatened, mainly because of habitat loss (Rudolf and Wesener 2017a-d).

This study is about a millipede collection of the Museum “La Specola”, the Natural History Museum of Florence located in central Italy, collected by Dr. Luca Bartolozzi and Dr. Stefano Taiti during two expeditions to Madagascar in 1989 and 1991. A total of 24 millipede species was identified, of which 17 are indigenous to Madagascar, and seven are introduced species. Among the seven introduced species, three are new records. New locality data is provided for eleven species, of which ten are indigenous. The most spectacular find was the presence of three undescribed giant pill-millipede species. Numerous additional specimens were also present, but species-level determination was impossible as they were females or immatures.

Here, we describe the three new species of endemic giant pill-millipedes of the genus *Zoosphaerium*. The three new species belong to the *Z. coquerelianum* species group, making it the most diverse species group with 22 representatives (Sagorny and Wesener 2017).

## Material and methods

### Abbreviations:

**MZUF**Museum "La Specola", Florence, Italy.

**ZFMK** Zoologisches Forschungsmuseum Alexander Koenig, Bonn, Germany

### Illustrations

The first and second right legs, ninth left leg, as well as the anterior and posterior telopods were dissected and drawn using a *camera lucida* mounted on an Olympus SZX12 stereo-microscope and later transferred to ink using Pigma Micron pens of widths 0.20 mm and 0.40 mm. For scanning electron microscopy (SEM) imaging, the right antenna and a small part of the endotergum from a mid-body tergite were dissected, cleaned, undergone a dehydration ethanol chain procedure (1 x 90%, 2 x 96%, 2 x 100%), then dried for 24 h, and mounted on aluminum stubs. The stubs with samples were coated with gold for 240 seconds in a sputter coater. SEM images were taken using a Supra VR 300VP (Carl Zeiss AG) scanning electron microscope utilizing the Software SmartSEM V05.00 based at the ZFMK. The SEM samples were returned to ethanol after the study. All ink drawings and images were edited using Adobe Photoshop CS2, later labelled and assembled into plates in Adobe Illustrator CS2.

### DNA extraction attempts

DNA extraction, amplification, and sequencing were conducted under identical conditions to those of earlier studies (Sagorny and Wesener 2017, Moritz and Wesener 2017, Wesener 2018), with the COI JJ primer (Astrin and Stüben 2008) being used for both PCR and sequencing. A translation into amino acids showed a similar composition to those of related species. Only a single sequence of one of the species *Zoosphaerium bartolozzii* sp. nov. (P_05) could be successfully sequenced due to the old age of the material. The sequence has been uploaded to GenBank under the accession number MN783351.

This one sequence was added to a fasta file containing COI sequences of all available *Zoosphaerium* sequences from GenBank (*N* = 14), as well as two sequences of the related Malagasy genus *Sphaeromimus*, as the near outgroup and a species of the unrelated family Procyliosomatidae from Australia as the far outgroup (Wesener and VandenSpiegel 2009, Wesener et al. 2010, 2014), bringing the total number of terminals to 18.

### Genetic analyses

Sequences were aligned by hand in Bioedit (Hall 1999). The final dataset consisted of 18 sequences and 657 base pairs. Pairwise distances: The number of base differences per site between sequences is shown in Table 1. The analysis involved 18 nucleotide sequences. Codon positions included were 1^st^+2^nd^+3^rd^. All ambiguous positions were removed for each sequence pair.

**Table 1.** Genetic *p*-distances between species of *Zoosphaerium* Pocock, 1895. Variation among sites was modeled with gamma distribution with shape parameter = 0.9659. Included were codon positions 1^st^+2^nd^+3^rd^.

*Procyliosoma* sp. (FJ409911.1)																	
*Sphaeromimus musicus* (KJ713245.1)	0.234																
*Sphaeromimus musicus* (KJ713244.1)	0.234	0.000															
*Zoosphaerium villosum* (KY399028.1)	0.228	0.180	0.180														
*Zoosphaerium* sp. green (KY399027.1)	0.237	0.191	0.191	0.116													
***Zoosphaerium mangabe* sp. nov. (KY399026.1)**	0.209	0.200	0.200	0.135	0.116												
*Zoosphaerium* sp. (KY399025.1)	0.237	0.197	0.197	0.047	0.135	0.136											
*Zoosphaerium* sp. (KY399024.1)	0. 239	0.199	0.199	0.046	0.047	0.135	0.001										
*Zoosphaerium minutus* (KY399023.1)	0.200	0.196	0.196	0.129	0.046	0.091	0.122	0.120									
*Zoosphaerium minutus* (KY399022.1)	0.208	0.193	0.193	0.131	0.129	0.095	0.123	0.122	0.012								
*Zoosphaerium bemanevika* (KY399021.1)	0.227	0.169	0.169	0.108	0.131	0.141	0.117	0.119	0.131	0.131							
*Zoosphaerium bemanevika* (KY399020.1)	0.231	0.172	0.172	0.110	0.108	0.144	0.114	0.116	0.131	0.134	0.010						
*Zoosphaerium bemanevika* (KY399019.1)	0.227	0.169	0.169	0.108	0.110	0.141	0.117	0.119	0.131	0.131	0.000	0.010					
*Zoosphaerium* sp. brown (FJ409931.1)	0.203	0.194	0.194	0.163	0.108	0.150	0.166	0.165	0.129	0.129	0.150	0.153	0.150				
*Zoosphaerium neptunus* (FJ409929.1)	0.208	0.194	0.194	0.151	0.163	0.148	0.154	0.153	0.139	0.145	0.150	0.156	0.150	0.151			
*Zoosphaerium alluaudi* (FJ409927.1)	0.202	0.171	0.171	0.148	0.151	0.131	0.151	0.153	0.120	0.120	0.151	0.156	0.151	0.114	0.148		
*Zoosphaerium alluaudi* (FJ409926.1)	0.208	0.178	0.178	0.151	0.148	0.141	0.160	0.162	0.129	0.129	0.160	0.165	0.160	0.123	0.157	0.013	
***Zoosphaerium bartolozzii* sp. nov. (P_05)**	0.215	0.199	0.199	0.144	0.151	0.131	0.139	0.141	0.122	0.119	0.150	0.151	0.150	0.134	0.147	0.113	0.122

The best-fitting substitution model for maximum-likelihood analysis was calculated with Model test (Tamura and Nei 1993) as implemented in MEGA6 (Tamura et al. 2013). The best-fitting model was the General Time Reversal (GTR)-Model (Tavaré 1986) with gamma distribution and Invariant sites (GTR+G+I) (lnL = -3600.757, Invariant = 0.50, Gamma = 0.9659, R = 4.09; Freq A: 0.30, T: 0.339, C: 0.204, G: 0.157).

**Figure 1.**
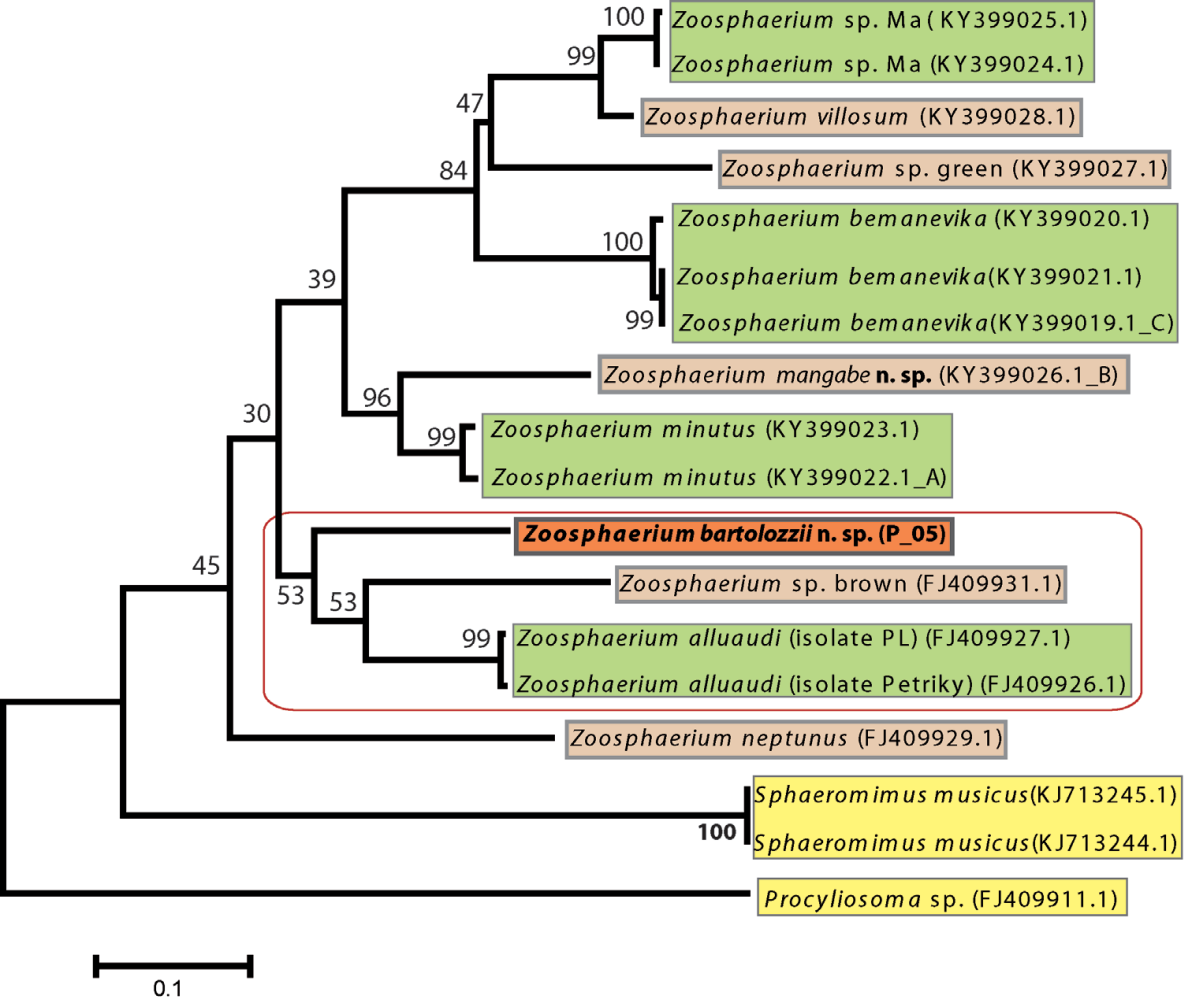
Maximum likelihood tree inferred from the COI dataset with 1000 bootstrap pseudoreplicates implementing the GTR+I+G model. Colors used to separate species. The circle indicates weakly supported sister-group relationships.

The evolutionary history was inferred by using the Maximum Likelihood method based on the General Time Reversible model. The tree with the highest log likelihood (-3590.0809) is shown in Fig. 1. The percentage of trees in which the associated taxa clustered together is shown next to the branches. Initial tree(s) for the heuristic search were obtained automatically by applying Neighbor-Joining and BioNJ algorithms to a matrix of pairwise distances estimated using the Maximum Composite Likelihood (MCL) approach, and then selecting the topology with superior log likelihood value. A discrete Gamma distribution was used to model evolutionary rate differences among sites (five categories (+G, parameter = 0.9659)). The rate variation model allowed for some sites to be evolutionarily invariable ([+I], 49.3492% sites). The tree is drawn to scale, with branch lengths measured in the number of substitutions per site. All positions with less than 5% site coverage were eliminated. That is, fewer than 95% alignment gaps, missing data, and ambiguous bases were allowed at any position. Evolutionary analyses were conducted in MEGA6.

## Results

### Genetic distances:

*Zoosphaerium bartolozzii* sp. nov. differs from all other analyzed species of the genus by a minimum of 11% uncorrected p-distance. The smallest genetic distances are shown towards *Z. alluaudi* (de Saussure & Zehntner, 1902) belonging to the *Z. coquerelianum* species group in which *Z. bartolozzii* sp. nov. is currently placed. Comparably low genetic distances of 11.9% are shown towards *Z. minutus* Sagorny & Wesener, 2017, which is currently not placed in any species group. A similar pattern is observed for *Z. mangabe* sp. nov. which shows a distance of 9.1–9.5% to *Z. minutus*. Genetic distances to other members of the *Z. coquerelianum* species group such as *Z. bemanevika* Sagorny & Wesener, 2017 and *Z. villosum* Wesener & Sierwald, 2005 are within 15%, similar to species belonging to different species groups such as *Z. neptunus*. In the phylogenetic tree, *Z. bartolozzii* sp. nov. is placed in a weakly supported clade together with an undescribed gigantic species from the Andohahela national park and *Z. alluaudi*.

### Taxonomy

#### Genus Zoosphaerium

Taxon classificationAnimaliaDiplopodaArthrosphaeridae

Pocock, 1895

26984DA0-25FA-5665-94CF-07D74DB9D731

See Wesener (2016) and Sagorny and Wesener (2017) for a recent catalogue and key to the species.

#### Zoosphaerium mangabe

Taxon classificationAnimaliaDiplopodaArthrosphaeridae

Wesener sp. nov.

5996D38D-0759-5124-B782-34DC94DAF9FC

http://zoobank.org/F9D03D6A-2AEB-4208-B64A-80554641BE6A

**Figure 2.**
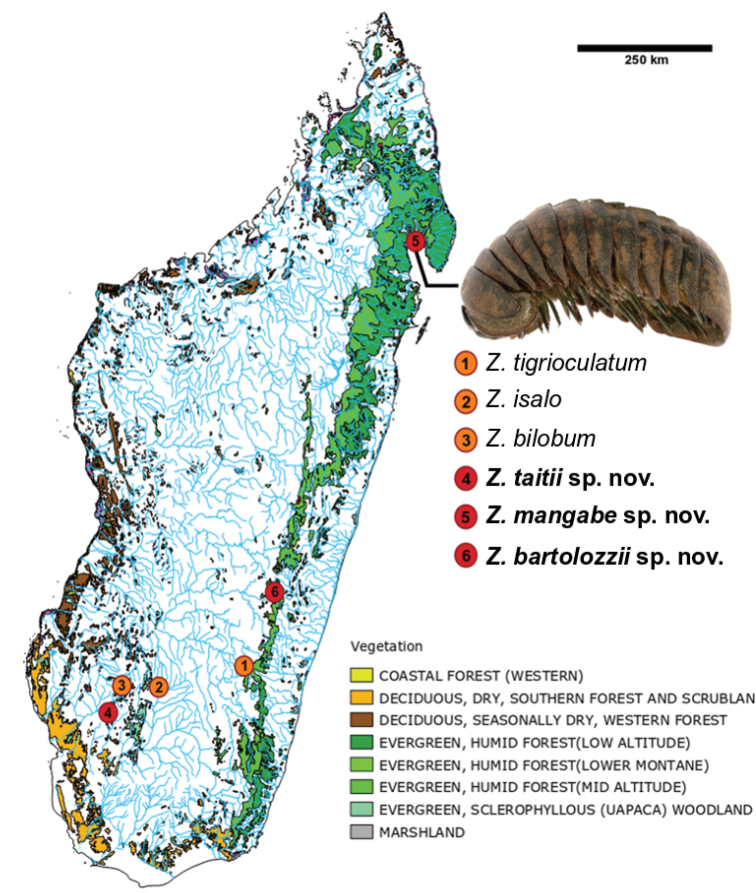
Distribution map of the three new *Zoosphaerium* species and the morphologically related species. Photograph shows the holotype of *Zoosphaerium mangabe* sp. nov., male.

**Figure 3.**
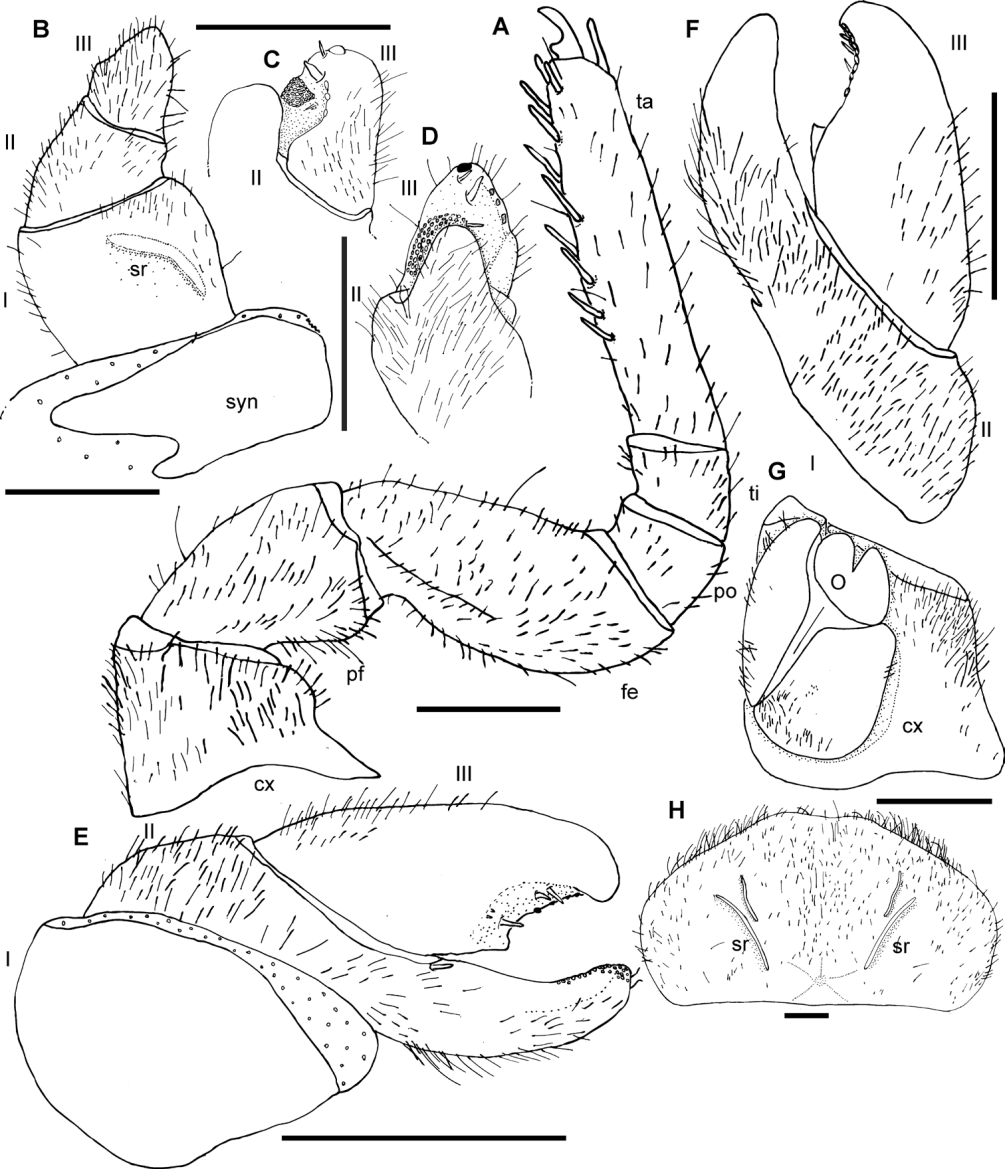
*Zoosphaerium mangabe* sp. nov., male holotype, female from Marojejy. **A** 9^th^ left leg **B**–**D** Left anterior telopod, **E**, **F** posterior telopod. **B** anterior view **C** lateral view **D** posterior view **E** anterior view **F** posterior view **G** female vulva **H** female washboard. Abbreviations: cx = coxa; fe = femur; O = operculum; pf = prefemur; po = postfemur; sr = stridulation rib; syn = syncoxite; ta = tarsus; ti = tibia; roman numerals refer to telopoditomere number. Scale bars: 1 mm.

Figures 2 , 3A–H , 5A

##### Material examined.

**1** ♂ ***holotype* (MZUF)**, Nosy Mangabe (Maroantsetra), 15°29'43.4''S, 49°46'07.6''E, Mag 1313, R. Nicheri, 24 Apr 1990.

##### Other material examined.

1 ♂ (**ZMUCXXXX**), Madagascar, Province Antsiranana, Marojejy Res., 8.4 km NNW Manantenina, 14°26'S, 49°45'E, 700 m, 10-16 Nov 1991, leg. J. Coddington, N. Scharff, S. Larcher, C. Griswold, R. Andriamasimanana; 1 ♂ (**ZFMK MYR8915**), same data as previous; 1 ♀ (**FMNH-INS 2858681B**), VS-2642, Madagascar, Antsiranana, SAVA, Parc National de Marojejy, 6.5 km NW Manantenina village, 14°27'21.2''S, 49°46'29.8''E, 780 m, disturbed lowland humid forest, pitfalls, coll. 31 May 2016, Voahangy Soarimalala, GenBank #KY399026.

##### Etymology.

The word *mangabe* is a noun in apposition, after the type locality of the species, the island of Nosy Mangabe at the NE coast of Madagascar.

##### Diagnosis.

*Zoosphaerium mangabe* sp. nov. shares the large body size, surface structure (like the peel of an orange), presence of only one stridulation rib on the male harp, and > 10 apical cones on the antenna only with *Z. coquerelianum* (de Saussure & Zehntner, 1897) and *Z. tainkintana* Wesener, 2009. *Zoosphaerium mangabe* sp. nov. differs from *Z. coquerelianum* in the long second locking carina on the anal shield (> times longer than the first), the hairy anal shield, and the presence of sclerotized teeth on the anterior telopods. The former differs from *Z. tainkintana* in the much shorter marginal bristles of the endotergum (reaching only 1/3 of the distance towards margin), the female operculum (two widely separated tips vs. fused tips), and in structures of the anterior telopod (e.g., three or four large teeth in *Z. mangabe* sp. nov. but seven in *Z. tainkintana*).

##### Description

(all measurements in mm). Body length: Male holotype: length 49.3, width 27.4 (2^nd^), 27.9 (8^th^ = widest), height 13.7 (2^nd^), 15.5 (8^th^ = highest). Female from Marojejy (broken): length ca. 50, width 27.9 (2^nd^ = widest), height 14.6 (2^nd^), 18.1 (8^th^ = highest).

*Coloration*: Color in some parts faded to a lighter brown than other parts after almost 30 years in ethanol. Younger and better-preserved female from Marojejy (FMNH-INS 2858681B) shows dark grey tergites with a thin dark brown posterior margin. Clypeus, base of legs and tip of antennae lighter brown, other parts of appendages dark green. Head except clypeus, collum, thoracic shield, body tergites, and anal shield dark olive green.

*Head*: Eyes consisting of 65/68 ommatidia. Antennae with 36/48 apical cones, part of left tip apparently regenerated.

*Gnathochilarium*: Sensory cones of palpi in single field. Inner parts of gnathochilarium not dissected.

*Mandible* not dissected.

*Stigmatic plate*: First stigmatic plate slender, apically narrow but well-rounded.

*Pleurite*: First pleurite laterally sharp-edged but not projecting.

*Collum*: Anterior and posterior margins with a sparse row of short setae . Inner part with a few isolated short setae.

*Thoracic shield*: Grooves deep, with few long setae. Remaining surface of thoracic shield similar to following tergites.

*Tergites*: Surface orange-like, each pit carrying a tiny seta. Tergite tips strongly projecting posteriorly.

*Endotergum*: Inner area with conical spines, broad at base with numerous setae and numerous small sharp spines in between. Single row of interchanging elliptical and smaller circular cuticular impressions. Smooth marginal ridge. Two rows of very short marginal bristle, protruding towards 1/4–1/2 margin (Fig. 5A).

*Anal shield*: Well-rounded, well-visible dorsally. Completely and regularly covered by small setae, underside carrying two locking carinae, second more than four times as long as first.

*Legs*: Leg one with three or five, leg two with six, leg three with seven ventral spines. First two leg pairs without an apical spine. Legs 4–21 with 8–10 ventral spines and one apical spine (Fig. 3A). In leg nine femur 1.9, tarsus 4.1 times longer than wide.

*Female sexual characters*: Vulva large, covering 3/4 of coxa, not extending to prefemur but protruding to apical margin of coxa. Operculum rounded, medially deeply invaginated, apical margin extended into two well-rounded lobes. Inner mesal plate long and slender and extending to apex of coxa and operculum. Lateral margin covered by hairs. External mesal plate broader and only extending to base of operculum, lateral margins also covered by hair (Fig. 3G).

*Subanal plate*: Large and wide, with shallow invagination at apical margin. Washboard with two short but well-developed stridulation ribs on each side. Margins and median part densely covered with hair (Fig. 3H).

*Male sexual characters*: Gonopore slightly oval, rounded apically, apical 1/4 covered by semicircular membranous plate, basal 3/4 by sclerotized plate, with few setae.

*Anterior telopod*: Harp carrying one stridulation rib positioned medially with end pointing laterad. Podomere one wide with few setae in anterior aspect (Fig. 3B). Podomere two, process not visible in anterior view, reaching half of length of podomere three (Fig. 3C, D), with sclerotized nubs along mesal margin. Podomere three tapering, as long as podomere two, with a rounded apex carrying one dark sclerotized spot near tip (Fig. 3C, D). Field of sclerotized spots run along apical-basal margin with three spines (Fig. 3C, D). Three or four crenulated teeth at lateral margin (Fig. 3C, D).

*Posterior telopod*: Movable finger 2.4 times longer than wide with tip slightly curving towards the immovable finger. Apical tip with ten sclerotized crenulated teeth, three spines, and a shallow mesal cavity with one triangular membranous lobe (Fig. 3E, F). Immovable finger basally with one spine (Fig. 3E); slender, 3.4 times longer than wide, reaching as far as movable finger, tip curved towards movable finger with a row of small sclerotized spots along apical part of mesal margin. Podomere one glabrous, podomere two in both aspects densely covered with setae, apical part of immovable finger glabrous. Movable finger with few setae in latero-basal part in both aspects (Fig. 3E, F).

##### Intraspecific variation.

Surprisingly, the specimens from Marojejy are in almost all aspects identical to the one studied from Nosy Mangabe. The genetic barcode comes from the female, and was previously published as "*Zoosphaerium* sp. Grey" (Sagorny and Wesener 2017).

#### Remarks

The following two new species are closely related to *Z. isalo* Wesener, 2009, *Z. bilobum* Wesener, 2009, and *Z. tigrioculatum* Wesener & Bespalova, 2010, of the *Z. coquerelianum* species group. All five species share the presence of a single stridulation rib on the male harp, four apical cones on the antenna, and, uniquely for species of the *Z. coquerelianum* species group, the presence of two instead of a single membranous lobe on the movable finger of the posterior telopod.

##### Determination key

**Table:** 

1	Process of second podomere of anterior telopod not visible in anterior view. Fifth antennomere with field of sensilla basiconica. Collum with isolated, long setae. Endotergum with row of large cuticular impressions and second row of much smaller impressions, bristles long, strongly protruding above tergite	***Z. bilobum* Wesener, 2009**
–	Process of second podomere of anterior telopod visible in anterior view	**2**
2	Podomere three of anterior telopod with crenulated teeth	**3**
–	Podomere three of anterior telopod without crenulated teeth	**4**
3	Collum glabrous with few setae at corners on either side of head. Endotergum with two rows of regularly distributed circular impressions, marginal bristles strongly protruding above tergite. Sensilla basiconica present on antennomere one and two. Anal shield weakly bell shaped	***Z. trigrioculatum* Wesener & Bespalova, 2010**
–	Collum glabrous, anterior margin with two rows of setae. Endotergum with single row of elliptical cuticular impression, marginal bristles protruding to margin. Sensilla basiconica absent. Anal shield well rounded	***Z. bartolozzii* sp. nov.**
4	Collum glabrous. Endotergum with single row of large cuticular impressions, marginal bristles slightly protruding above tergite. 2^nd^ leg with four or five ventral spines. Anal shield tapering	***Z. isalo* Wesener, 2009**
–	Collum glabrous. Endotergum with single row of slightly rounded elliptical cuticular impressions, marginal bristle protruding to margin. 2^nd^ leg with six or seven ventral spine. Anal shield well rounded.	***Z. taitii* sp. nov.**

##### Zoosphaerium bartolozzii

Taxon classificationAnimaliaDiplopodaArthrosphaeridae

Anilkumar & Wesener sp. nov.

10A06356-DC3E-5975-BA8B-96782EF20BF8

http://zoobank.org/15D3E6C0-73DE-4939-B3E3-AEB577711707

**Figure 4.**
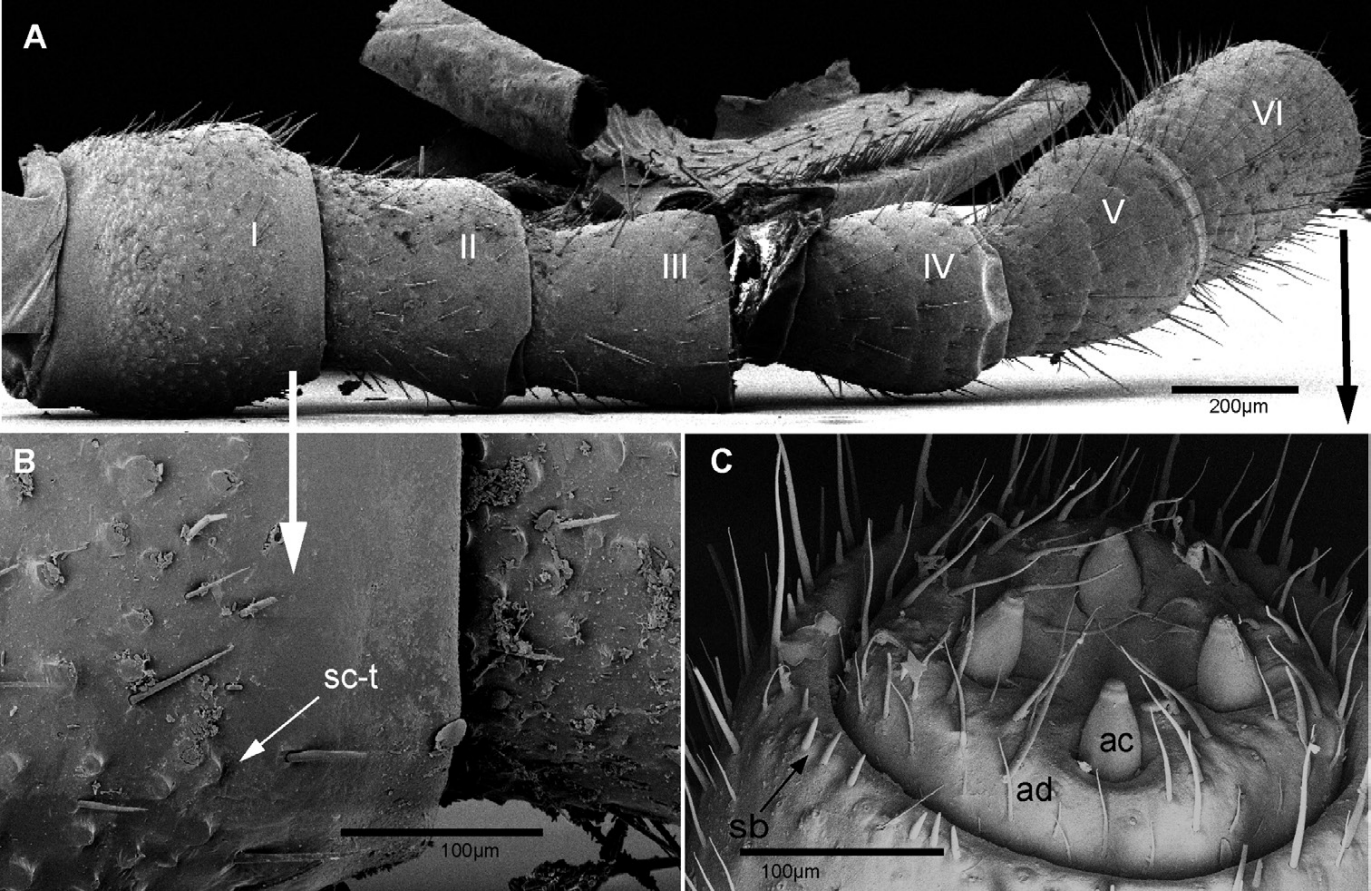
*Zoosphaerium bartolozzii* sp. nov., male holotype, SEM, Right antenna. **A** lateral view **B** antennomeres 1 and 2 with sclerotized teeth **C** apical disc with four sensory cones. Abbreviations: ac = apical cone; ad = apical disc; sb = sensilla basiconica; sc-t = sclerotized teeth.

**Figure 5.**
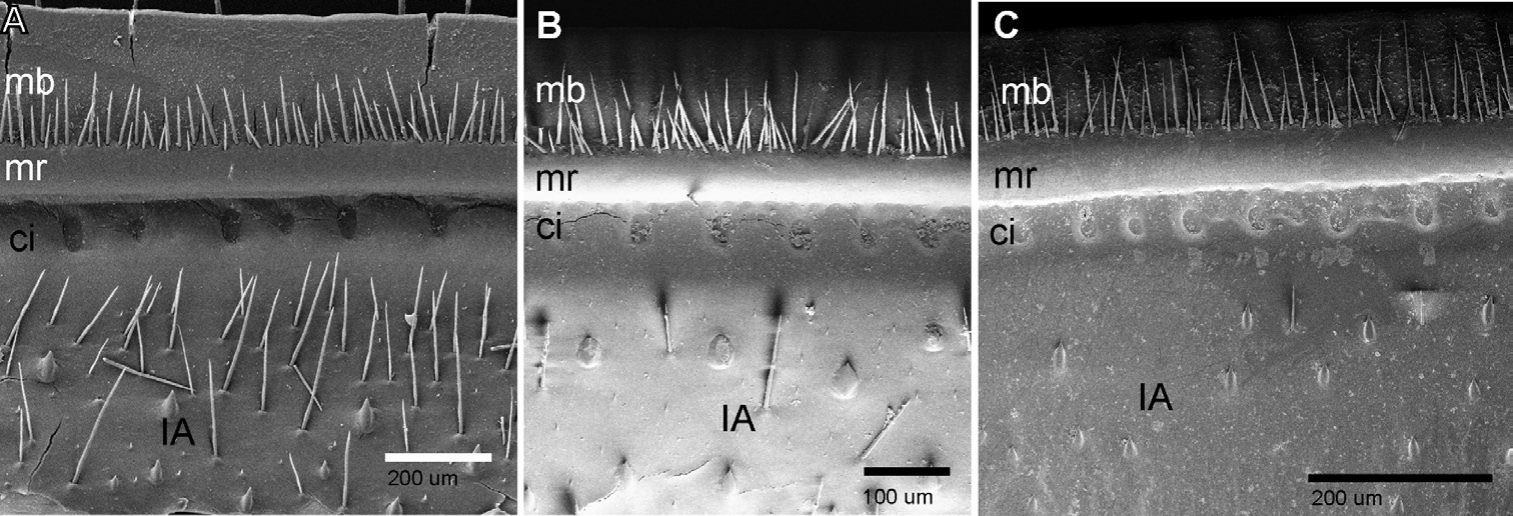
SEM, Endotergum of mid body tergites, ventral view. **A***Zoosphaerium mangabe* sp. nov., male from Marojejy **B***Zoosphaerium bartolozzii* sp. nov., male holotype **C***Zoosphaerium taitii* sp. nov., male holotype. Abbreviations: IA = inner area; ci = cuticular impressions; mr = marginal ridge; mb = marginal bristles.

**Figure 6.**
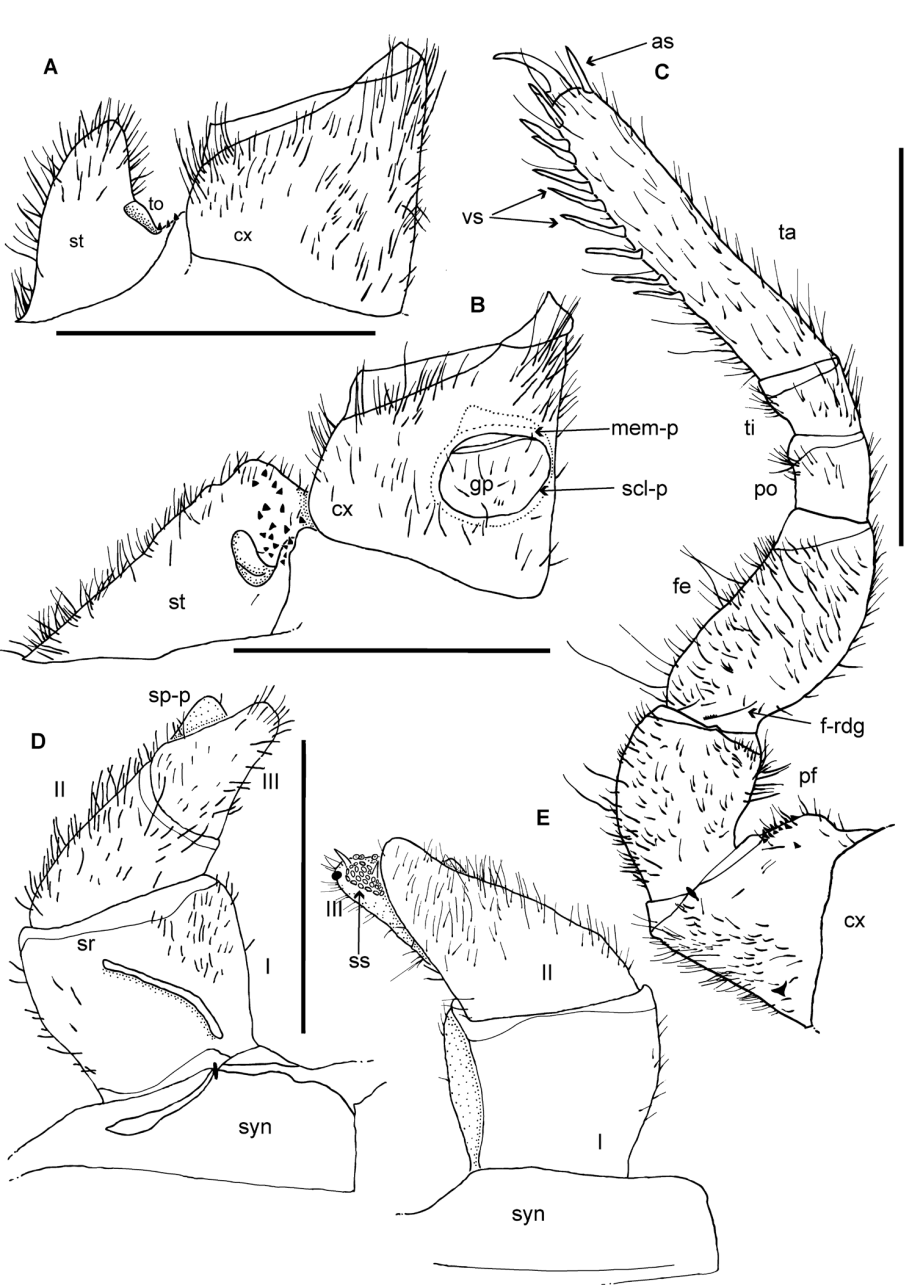
*Zoosphaerium bartolozzii* sp. nov., male holotype. **A**, **B** Coxae of first and second right legs, **D**, **E** Left anterior telopod. **A** first stigmatic plate **B** second stigmatic plate **C** 9^th^ left leg **D** anterior view **E** posterior view. Abbreviations: as = apical spine; cx = coxa; fe = femur; f-rdg = femur ridge; gp = gonopore; mem-p = apical membranous part of plate covering gonopore; pf = prefemur; po = postfemur; scl-p = sclerotized plate; sp-p = second podomere process; sr = stridulation rib; ss = sclerotized spot; st = stigmatic plate; syn = syncoxite; ta = tarsus; ti = tibia; to = tracheal opening. Scale bars: 1 mm.

**Figure 7.**
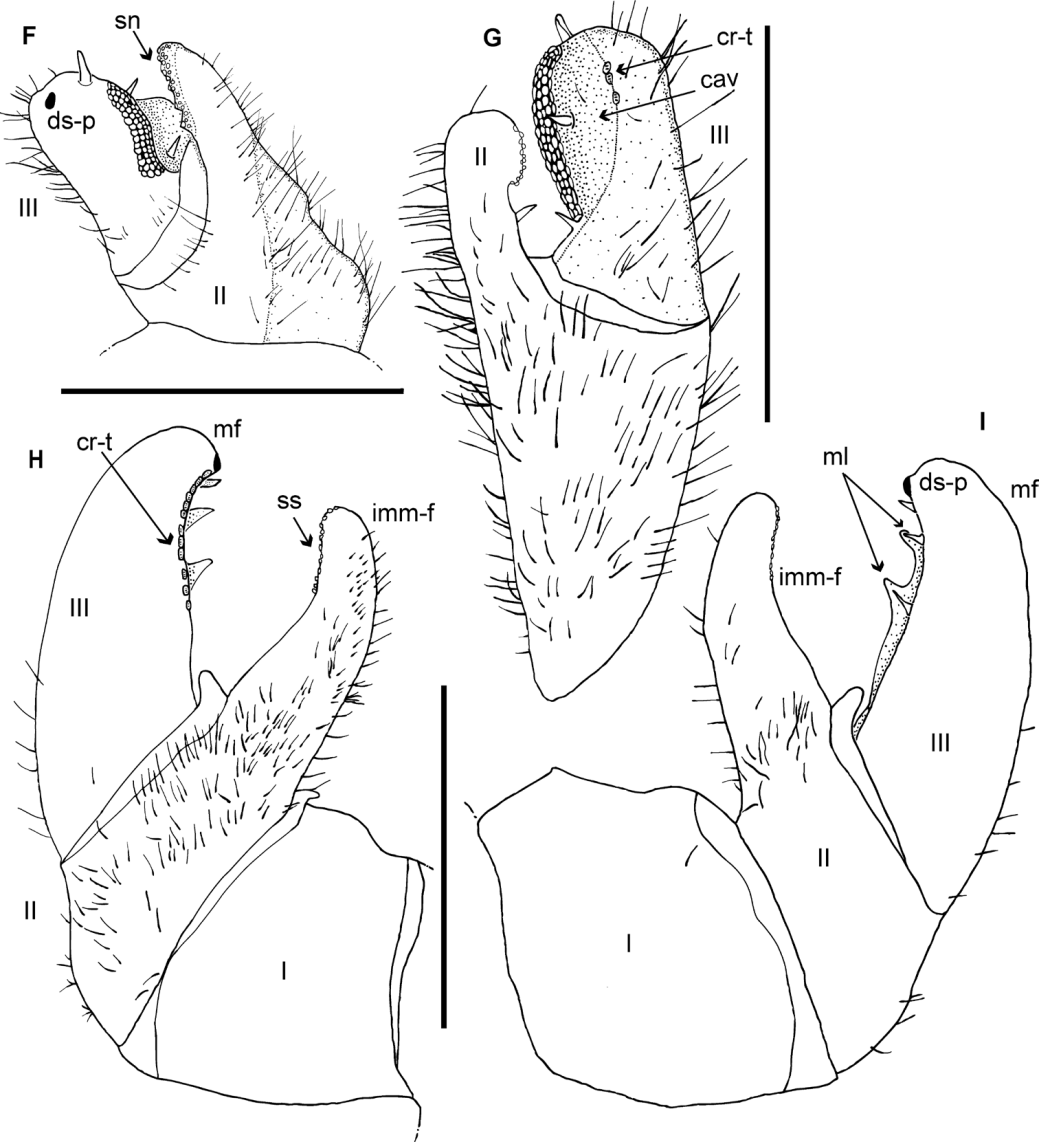
*Zoosphaerium bartolozzii* sp. nov., male holotype. **F**, **G** Left anterior telopod, **H**, **I** posterior telopod. **F** mesal view **G** lateral view **H** posterior view **I** anterior view. Abbreviations: cav = cavity; cr-t = crenulated teeth; ds-p = dark sclerotized spot; imm-f = immovable finger; mf = movable finger; ml = membranous lobes; sn = sclerotized nubs; **ss** = sclerotized spot. Scale bars: 1 mm.

Figures 4 , 5B , 6 , 7

###### Etymology.

Adjective, the species is named after the Italian beetle expert Dr. Luca Bartolozzi who collected this species.

###### Material examined.

**1** ♂ ***Holotype* (MZUF)**, Madagascar: 5 km S di Ambalamanakana, (strada Ambositra – Fianarantsoa) in forests, 20°46'49.2"S, 47°10'48.5"E, n. Mag. 1107. Legit: Bartolozzi, S. Taiti, C. Raharimina, 10 May 1991.

###### Diagnosis.

*Zoosphaerium bartolozzii* sp. nov. is most similar to *Z. tigrioculatum* due to the presence of three sclerotized crenulated teeth on the podomere three of the anterior telopod, and also in the visibility of the process of the 2^nd^ podomere in anterior view (Figs 6D, 7G). *Zoosphaerium bartolozzii* sp. nov. differs from *Z. tigrioculatum* in the presence of a single row cuticular impression on the endotergum (two rows in the latter), the absence of sensilla basiconica on antennomeres one and two, and the presence of a well-rounded anal shield which is slightly bell-shaped in *Z. tigrioculatum*.

###### Description

(all measurements in mm):

Body length: holotype male: length 24.2, width 11 (2^nd^ = widest), height 6.2 (2^nd^ = highest).

*Coloration*: Faded due to 27 years of preservation in alcohol. Legs and antennae dark green. Head and collum dark olive-green. Tergites and anal shield faded dark green-brown.

*Head*: Eyes with 90–100 ommatidia. Antennae long and protruding up to leg pair six. Size of antennomeres 1>2<3=4<5<6 (Fig. 4A). Antennomere 1 broad, antennomeres 1–3 with large rounded protuberant sclerotized teeth (Fig. 4B). Antennomeres 3–6 covered with large setae. Antennomere 6 with a single row of sensilla basiconica surrounding the apical disc, with four apical sensory cones (Fig. 4C).

*Gnathochilarium*: Lateral stipites and central mentum with long setae, setae absent at center of lamellae linguales. Inner palpi protruding to medial side of gnathochilarium bearing single field of sensory cones. Rudimentary lateral palpi sharing a well-developed base bearing four sensory cones. Hypopharynx with single row of marginal teeth. Central pads apically protruding from lamellae linguales, with a median triangular incision on each pad. Posterior half of underside with single field of large sensory cones interspersed with longer, slimmer structures.

*Mandible* not dissected.

*Stigmatic plates*: First stigmatic plate triangular, with marginal setae and some extra setae at elliptical apex, three spines near tracheal opening (Fig. 6A). Second stigmatic plate triangular, with a slightly curved apex. Marginal setae dense at base, 19 spines near tracheal opening (Fig. 6B).

*Pleurite*: First pleurite with a rounded tip protruding backwards.

*Collum*: Surface glabrous, anterior margin with two rows of setae. Posterior margin laterally with few isolated setae.

*Thoracic shield*: Lateral grooves shallow, setae only present in lateral grooves.

*Tergites*: Surface glabrous and slightly chagrined. Tips of paratergites slightly extending posteriorly.

*Endotergum*: Inner area with conical spines, broad at base with few setae and numerous small sharp spines in between. Single row of elliptical cuticular impressions. Smooth marginal ridge. Two rows of marginal bristles, majority protruding 1/4–1/2, a few to 3/4 of distance to margin (Fig. 5B).

*Anal shield*: Large and well rounded, completely covered with tiny setae, underside carrying two locking carinae, second 3.5 times longer than first.

*Legs*: Leg 1 with three or four, leg 2 with six or seven, leg 3 with seven ventral spines. Legs 1 and 2 without an apical spine. Legs 4–21 with eleven ventral spines and one apical spine (Fig. 6C). In leg 9 femur 1.7, tarsus 5.4 times longer than wide. Coxa with few spines. Femur ridge present. All podomeres covered with setae.

*Female unknown*.

*Male sexual characters*: Gonopore rounded, slightly divided near to apex, covered by 1/4 membranous plate apically and 3/4 sclerotized plate basally with few setae. Gonopore covering 1/4 height and 1/2 width of coxa (Fig. 6C).

*Anterior telopod*: Harp carrying one stridulation rib. Podomere 1 with few marginal and apical setae (Fig. 6D), and a shallow mesal cavity laterally (Fig. 6E). Podomere 2, process visible in anterior view, reaching 2/3^rd^ of length of podomere 3 (Fig. 6D). Podomere 2 process slightly slender apically, with sclerotized nubs along apical-mesal margin and a basal spine present below field of spots (Fig. 7F, G). Podomere 3 apically wide, visible as small triangular lobe in anterior aspect (Fig. 6D), with one dark sclerotized spot near apical margin (Figs 6E, 7F) and a broad mesal cavity with sclerotized spots running along apical-basal margin, with four spines and three sclerotized crenulated teeth at meso-apical margin of cavity (Fig. 7F, G). Two spines merged at apical margin above field of spots, one at center of cavity with tip protruding to sclerotized spots, one basal spine below field of spots. Podomeres 2 and 3 covered with setae (Fig. 7F, G).

*Posterior telopod*: Movable finger thicker (2.5 times longer than wide) and slightly longer than immovable finger, carrying one spine just below dark sclerotized spot along apical margin (Fig. 7H). Hollowed-out margin with two membranous lobes, each with one marginal spine centrally (Fig. 7I). 12 sclerotized crenulated teeth present marginally. Six teeth apically positioned together, three at center of margin (between two membranous lobes), and last three separated by a short distance, two directly at base and one near base of posterior membranous lobe (Fig. 7H). Movable finger with few basal marginal setae. Tips of podomeres 2 and 3 slightly curved towards one another. Immovable finger slender (3.2 times longer than wide) with sclerotized spots running from apical to mid margin. Immovable finger covered with setae in posterior aspect, one membranous lobe present between podomeres 2 and 3. Podomere 1 large, rectangular with no setae in anterior or posterior aspect (Fig. 7H, I).

##### Zoosphaerium taitii

Taxon classificationAnimaliaDiplopodaArthrosphaeridae

Anilkumar & Wesener sp. nov.

5D5FAC5D-AF07-518C-A816-410B129D7C33

http://zoobank.org/DA735EC5-8612-4C13-A42E-910718F6ED4C

**Figure 8.**
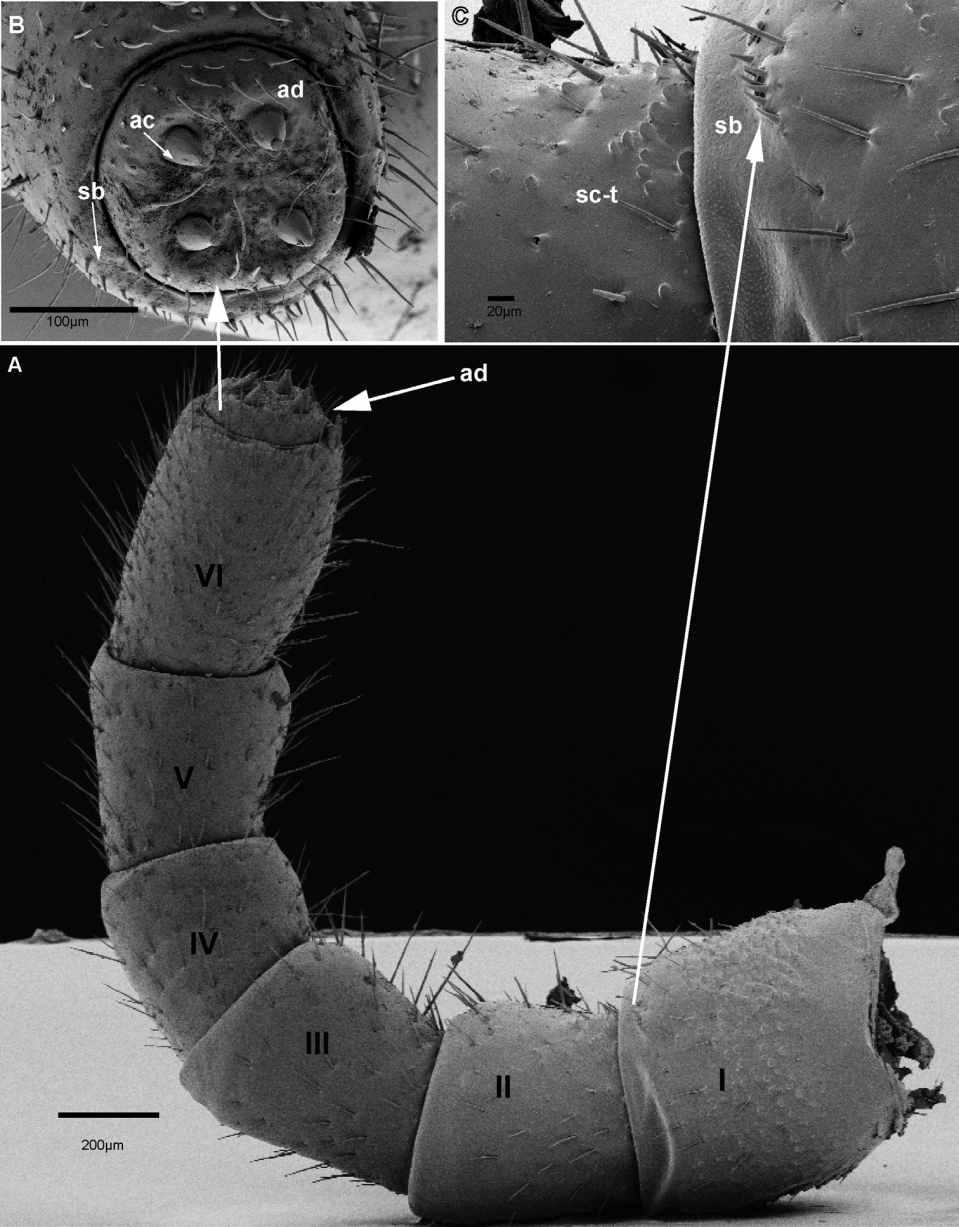
*Zoosphaerium taitii* sp. nov., male holotype, SEM, Right antenna. **A** lateral view **B** antennomeres 1 and 2 with sclerotized teeth **C** apical disc with four sensory cones. Abbreviations: ac = apical cone; ad = apical disc; sb = sensilla basiconica; sc-t = sclerotized teeth.

**Figure 9.**
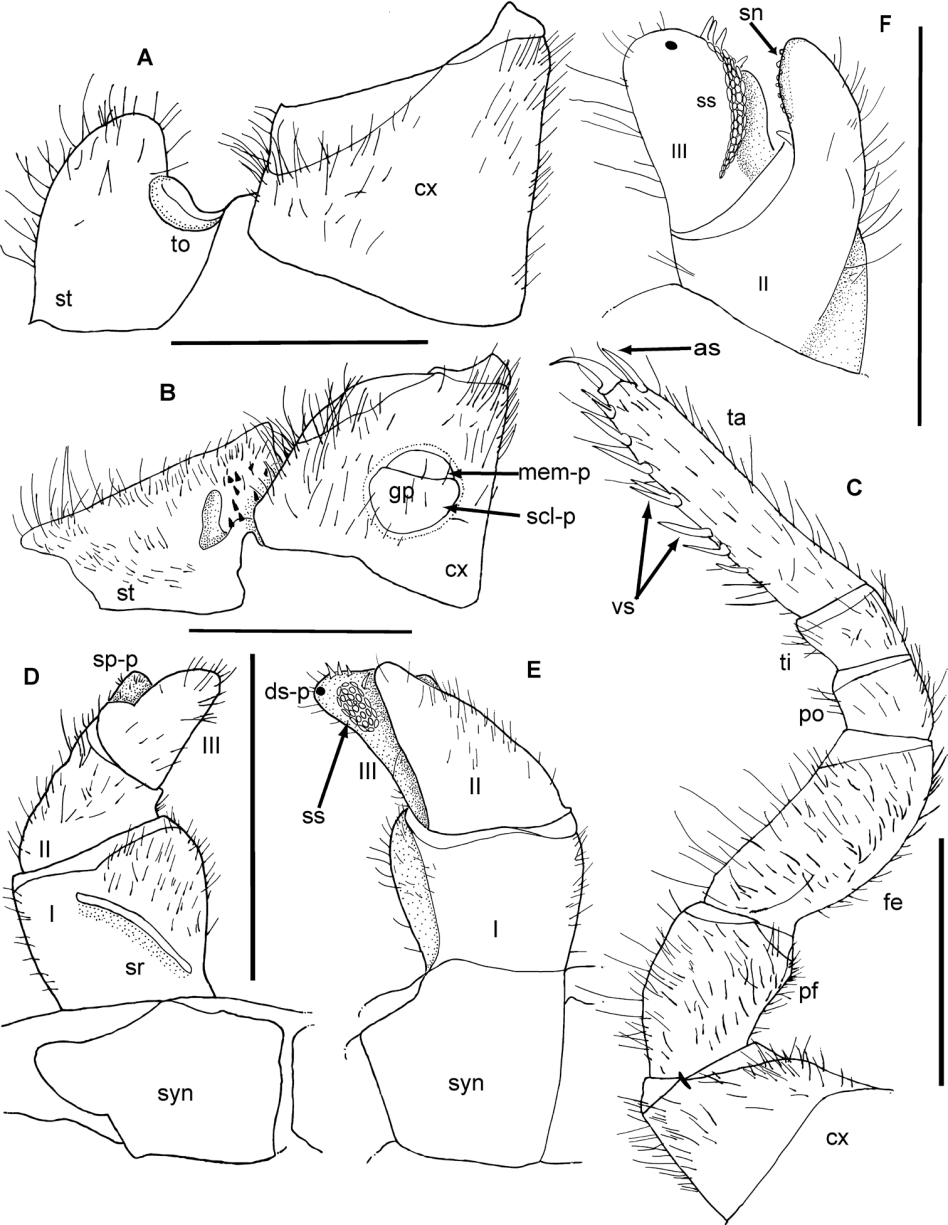
*Zoosphaerium taitii* sp. nov., male holotype. **A**, **B** Coxae of first and second right legs, **D**–**F** Left anterior telopod. **A** first stigmatic plate **B** second stigmatic plate **C** 9^th^ left leg **D** anterior view **E** posterior view **F** mesal view. Abbreviations: as = apical spine; cx = coxa; ds-p = dark sclerotized spot; fe = femur; f-rdg = femur ridge; gp = gonopore; mem-p = apical membranous part of plate covering gonopore; pf = prefemur; po = postfemur; scl-p = sclerotized plate; sp-p = second podomere process; sr = stridulation rib; ss = sclerotized spot; st = stigmatic plate; syn = syncoxite; ta = tarsus; ti = tibia; to = tracheal opening. Scale bars: 1 mm.

**Figure 10.**
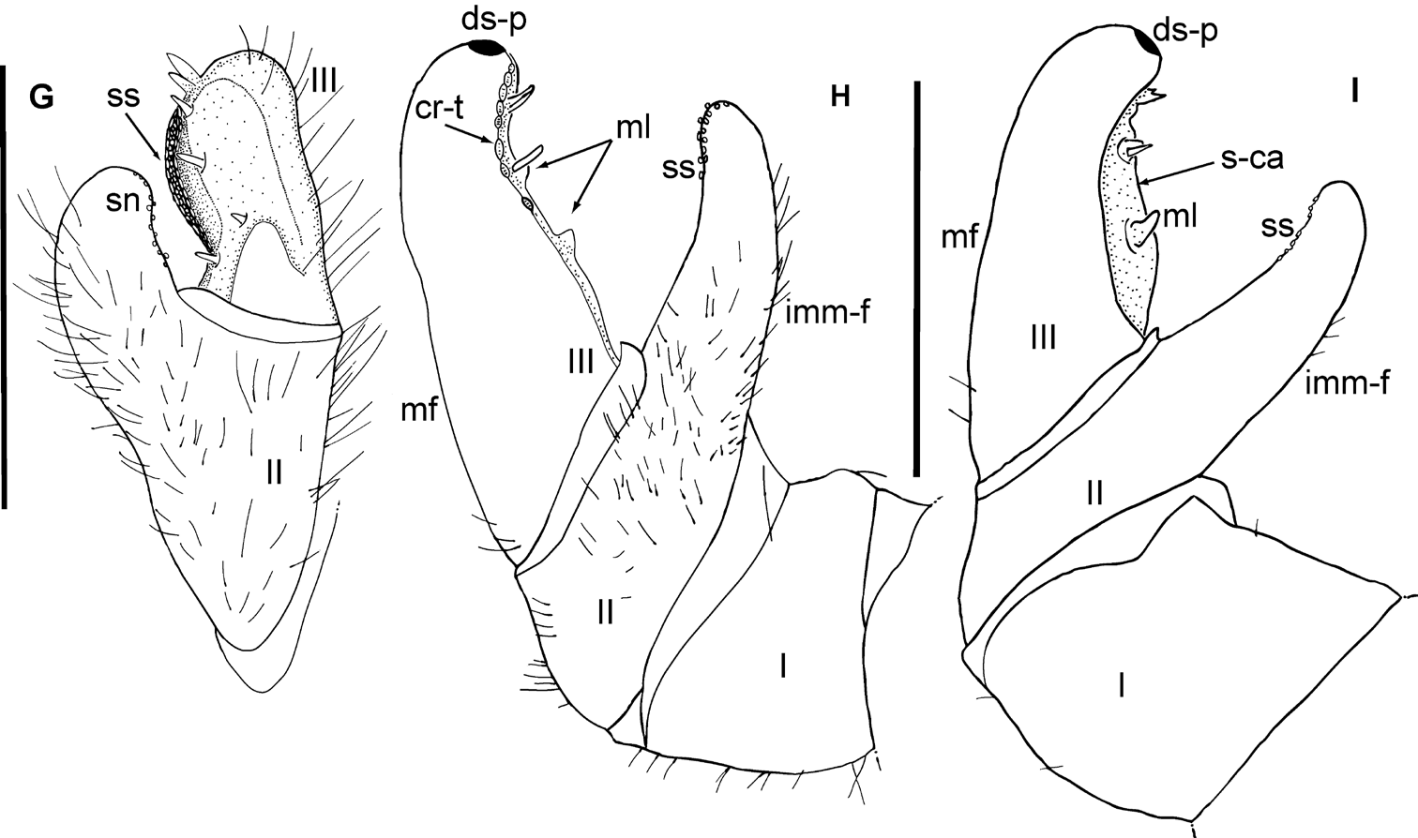
*Zoosphaerium taitii* sp. nov., male holotype, left anterior telopod. **G** lateral view. **H**, **I** Left posterior telopod. **H** posterior view **I** anterior view. Abbreviations: cr-t = crenulated teeth; ds-p = dark sclerotized spot; imm-f = immovable finger; mf = movable finger; ml = membranous lobes; s-ca = shallow cavity sn = sclerotized nubs; ss = sclerotized spot. Scale bars: 1 mm.

Figures 5C , 8 , 9 , 10

###### Etymology.

Adjective, the species is named after the land isopod expert Dr. Stefano Taiti who collected this species.

###### Material examined.

**1** ♂ ***Holotype* (MZUF)**, Madagascar: SW 17 km Edi Sakaraha, forêt de Zombitsy, foresta secca, 22°52'47.1"S, 44°36'41.1"E, n. Mag. 1107. Legit: Bartolozzi, S. Taiti, C. Raharimina, 15 May 1991.

###### Other material.

1 ♂, **CAS BLF Mei-99 Ma-14**, Province Toliara, Zombitse Nature Reserve, 16 km E Sakaraha, 825 m, tropical forest on sand, 22.88231°S, 44.70062°E, coll. E. L. Schlinger, M. E. Irwin, 15–18 Dec 1999.

###### Diagnosis.

*Zoosphaerium taitii* sp. nov. is mostly similar to *Z. isalo*, both differing from all other species in the anterior telopod where sclerotized teeth are absent on the third podomere. *Zoosphaerium taitii* sp. nov. differs from *Z. isalo* in the shorter marginal bristles of the endotergum (protruding above the tergite margin in *Z. isalo*), the higher number of ventral spines on leg 2 (four or five versus six or seven) and the slightly differently shaped anal shield (tapering in *Z. isalo*, well-rounded in *Z. taitii* sp. nov.).

###### Description

(all measurements in mm):

Body length: holotype male: length 20.4, width 9.4 (2^nd^) up to 9.9 (tergite 9 = widest), height 5.4 (2^nd^ = highest).

*Coloration*: Strongly faded due to exposure to alcohol. Antennae dark green. Legs basally brown and apically green. Head and collum light green. Tergites and anal shield faded light brown.

*Head*: Eyes with 60–70 ommatidia. Antennae short, protruding up to leg 3 or 4. Size of antennomeres 1>2<3>4<5<6 (Fig. 8A). Antennomeres 1–3 with sclerotized teeth. Antennomeres 1 and 6 with a single row of sensilla basiconica (Fig. 8B, C). Antennomeres 3–6 with long setae. Antennomere 6 with an apical disc containing four apical sensory cones (Fig. 8B).

*Gnathochilarium*: Stipites and central mentum with long setae, setae absent at center of lamellae linguales. Inner palpi protruding to medial side of gnathochilarium, bearing single field of sensory cones. Rudimentary lateral palpi sharing a well-developed base bearing four sensory cones. Hypopharynx with one row of marginal teeth. Central pads apically protruding from lamellae linguales, with a median triangular incision on each pad. Posterior half of underside with single field of large sensory cones interspersed with longer, slimmer structures.

*Mandible* not dissected.

*Stigmatic plates*: First stigmatic plate apically elliptical with marginal setae, lateral end pointed (Fig. 9A). Second stigmatic plate trapezoidal with nine spines near tracheal opening, covered with tiny setae inside and few long marginal setae (Fig. 9B).

*Pleurite*: First pleurite weakly extending posteriorly with a well-rounded tip.

*Collum*: Glabrous, anterior and posterior margin with sparse rows of isolated setae.

*Thoracic shield*: Glabrous expect for narrow lateral grooves.

*Tergites*: Surface glabrous and shiny, chagrined. Paratergite tips not projecting.

*Endotergum*: Inner area with narrow conical spines, very few isolated setae. A single row of rounded-elliptical cuticular impressions. Broad smooth marginal ridge. Two rows of marginal bristle protruding towards marginal brim, few reaching tip, other few reaching 1/4–3/4 of distance to margin (Fig. 5C).

*Anal shield*: Large and well rounded, surface glabrous. Two locking carinae, second carina 2.3 times longer than first, close to anal shield margin.

*Legs*: Leg 1 with four or five spines, leg 2 with six or seven spines, leg 3 with seven or eight ventral spines and an apical spine, legs 4–21 with nine ventral spines and one apical spine. In leg 9 femur 2.0, tarsus 4.7 times longer than wide. Uniform distribution of setae on all podomeres. Prefemur and femur with few long setae. Femur ridge length reaching 1/4 of femur length (Fig. 9C).

*Female unknown*.

*Male sexual characters*: Gonopore slightly oval, rounded apically, divided, reaching 1/2 length and 1/4 width of coxa, covered by 1/4 semicircular membranous plate apically and 3/4 sclerotized plate basally with few setae (Fig. 9B).

*Anterior telopod*: Harp carrying one stridulation rib positioned medially with one end pointing laterad. Podomere 1 broad with marginal setae, few setae above stridulation rib (Fig. 9D) and a shallow mesal cavity laterally (Fig. 9E). Podomere 2, process visible in anterior view, reaching 2/3^rd^ of length of podomere 3 (Fig. 9D), with sclerotized nubs along apical-mesal margin and one basal spine present below field of spots (Fig. 9F). Podomere 3 apically broad, rounded, longer than podomere 2 (Figs 9F, 10G), and carrying one dark sclerotized spot at apical tip (Fig. 9F). Field of sclerotized spots run along apical-basal margin with four spines out of six visible (Figs 9F, 10G). Three spines positioned at apical margin above field of spots, one in middle of cavity with tip protruding to sclerotized spot, one small single spine in lateral projected area slightly towards base, and one spine at base of field of spots (Fig. 10G). Podomeres 2 and 3 covered with setae except mesally (Fig. 10G).

*Posterior telopod*: Movable finger 2.5 times longer than wide with tip slightly curving towards immovable finger. Apical tip with a dark sclerotized spot, eight sclerotized crenulated teeth (arranged in three groups), three mesal spines (two merged at tip), with very few setae at base and a shallow mesal cavity with two membranous lobes (Fig. 10H, I). First five teeth positioned below apically merged spines, other two teeth below middle spine, located in middle of first membranous lobe, two isolated single tooth located between both membranous lobes (Fig. 10H). Immovable finger 4.3 times longer than wide, reaching 3/4^th^ of length of movable finger, slender, tip curved towards movable finger with a row of small sclerotized spots along 1/4 apical mesal margin, covered with setae in posterior aspect (Fig. 10H). Podomere 1 glabrous except for very few marginal setae.

###### Remarks.

This species was described as a population of *Z. isalo* in a previous study, already with a remark that the status of the population should be evaluated when more male specimens become available (Wesener 2009). With the additional male specimen available from the collections of the "La Specola" Museum, we feel more confident in describing the Zombitse specimens as a species separate from *Z. isalo*. The species lives in sympatry with *Z. album* Wesener, 2009, a species belonging to a different species group (Wesener 2016).

**Table 2.** Comparison of *Z. isalo* Wesener, 2009, *Z. bilobum* Wesener, 2009, *Z. tigrioculatum* Wesener & Bespalova, 2010, *Z. bartolozzii* sp. nov., and *Z. taitii* sp. nov. Abbreviations: ANT – Antenna; aT – anterior telopod, bas – basiconica, Endo – endotergum. Modified after Wesener et al. 2010b.

**Character**	***Z. isalo***	***Z. bilobum***	***Z. tigrioculatum***	***Z. bartolozzii* sp. nov.**	***Z. taitii* sp. nov.**
**Shape of anal shield**	Tapering	Tapering	Weakly bell shaped	Well rounded	Well rounded
**Locking carinae**	2^nd^ 3×1^st^	2^nd^ 2.5×1^st^	2^nd^ 4×1^st^	2^nd^ 3.5 ×1^st^	2^nd^ 2.3× 1^st^
**1^st^ leg no. of ventral spines**	3 or 4	6 or 7	4 or 5	3 or 4	4 or 5
**2^nd^ leg no. of ventral spines**	4 or 5	8 or 9	6 or 7	6 or 7	6 or 7
**aT, 2^nd^ podomere in av**	visible	Not visible	visible	visible	visible
**aT, 3^rd^ podomere in av**	without crenulated teeth	without crenulated teeth	with crenulated teeth	with crenulated teeth	without crenulated teeth
**ANT, sclerotized teeth**	on antennomeres 1-4	on antennomeres 1-5	on antennomeres 1-3	on antennomeres 1-3	on antennomeres 1-3
**ANT, sensilla bas.**	only on 1^st^	only on 1^st^ and 5^th^	on 1^st^ and 2^nd^	absent	only on 1^st^
**Endo, marginal bristle**	protruding slightly above margin	extending beyond margin	extending beyond	protruding to margin	protruding to margin
**Endo, cuticular patterns**	single row	two rows	two rows	single row	single row

#### New locality data

**Order Polyxenida**

Polyxenidae sp.

1; **Fi-xx**; Col des Tapia, fra Ambositra e Antsirabe, 1400 m, foresta di Tapia (Uapaca bojeri), 9 May 1991.

**Order Sphaerotheriida**

*Zoosphaerium neptunus* (Butler, 1872)

7 immatures; **Fi-19B**; Perinet, 29 May 1991 (foresta pluviale).

**Remarks**: This species is known to show swarming behavior near Perinet/Andasibe (Wesener and Schütte 2010).

*Zoosphaerium villosum* Wesener & Sierwald, 2005

1 M, 2 F; **Fi-01A**; Madagascar, Stat. For. Tampolo, 10 km N. Fenerive, foresta costiera, 1 Jun 1991.

*Zoosphaerium blandum* (de Saussure & Zehntner, 1897)

2 F; **Fi-03A**; Andohahela pII, foresta secca, 26 May 1991.

Zoosphaerium cf. pseudoblandum Wesener, 2009

2 F; **Fi-06B**; Andohahela pI, versante E, NW Ft. Dauphin, ca. 300 m, foresta pluviale, lettiera vagliata, 24-26 May 1991. 4 immatures; **Fi-24F**; RNI Andohahela, pI, versante E, ca. 300 m, lettiera vagliata, 24-26 May 1991.

Zoosphaerium cf. aureum Wesener, 2009

Juveniles; **Fi-Mag1058**; Mt d'Ambre, 1000-1200 m, 24 Sep 1989.

Zoosphaerium cf. album Wesener, 2009

2 F; **Fi-08A**; 17 km E. Sakaraha, Zombitsy, 15 May 1991.

*Sphaeromimus musicus* (de Saussure & Zehntner, 1897)

2 M; **Fi-02**; Ifaty, 20 km N. di Tulear, sotto corteccia, 16 May 1991.

**Remarks**: The following three species are distinct from any described ones, but cannot be formally named because no mature males are known.

*Zoosphaerium* sp. 1

2 F, 2 immatures; **Fi Mag 1058**; Tsaramandroso, Ankarafantsika, 13 Sept 1989.

*Zoosphaerium* sp. 2

2 F, 2 immatures; **Fi-05A**; PN Ranomafana, foresta, 11 May1991. 1 F; **Fi-X**; Ranomafana, NE Fianarantsoa, 950-1100 m, ettiera e sotto tranchi, 11-12 May 1991. 1 immature M; **Fi-Y**; Ranomafana, foresta secondaria, 1100 m, 12 May 1991.

*Zoosphaerium* sp. 3

1 F; **Fi-xx**; Mt d'Ambre, 1000 m, 23 Sept 1989.

*Zoosphaerium* spp.

Juveniles; **Fi-07D**; Andohahela pI, versante E, NW Ft. Dauphin, ca. 300 m, foresta pluviale, lettiera vagliata, 24-26 May 1991. 2 immatures; **Fi-xx**. Mt. d'Ambre 1000 m, 25 Sept 1989. 7 immatures; **Fi-29A**; Mt. d'Ambre 1100 m, 25 Sept 1989. 5 immatures; **Fi-11A**; Andohahela, pI, 500-600 m, foresta lettiera vagliata, 25 May 1991. 3 immatures; **Fi-14B**; Mahavelona (= Foulpointe), N. die Tamatave, foresta litorale, lettiera vagliata, 31 May 1991. 2 immatures; **Fi-xx**; Marojejy 1200 m, 28 Sept1989. 5 immatures; **Fi-32C**; Perinet, 1000 m, 8 Oct1989. 2 immatures F; **Fi-20A**; Manjakatompo, c/o station Pisciculture, 1700 m, 5 Oct1989. 1 immature; **Fi-31A**; 7 km NE di Ankaramena, SW di Ambalavao, boschetto di manghi lungo un torrente, 13 May1991. 5 immatures; **Fi-37D**; Montagne d'Andrangoatra (a N. diSambava), 29 Sept1989.

**Order Polyzoniida**

*Rhinotus purpureus* (Pocock, 1894)

6?; **Fi-35B**; Ranomafana, sotto cortecce di alteri morti, giardini, 12 May1991. 3?; **Fi-17D**; Nosy Be, spioggia Ambatoloaka, 15. Sept1989. 5?; **Fi-25B**; Nosy Be, c/o Cascata, 18 Sept1989. 5?; **Fi-23A**; Tampolo, foresta costiera. 12?; **Fi-15B**; Mahavelona (=Foulpointe), N. die Tamatave, foresta litorale, lettiera vagliata, 31 May1991. 4?; **Fi-18B**; Valle del Sambirano, 10 km SE Ambanja, 21 Sept1989. 1?; **Fi-32D**; Perinet, 1000 m, 8 Oct1989.

**Remarks**: This introduced species is very common in humid forests on Madagascar. Potential indigenous species of other siphonotid genera also exist, but are rare and unnamed (Wesener 2014a).

**Order Siphonophorida**

*Siphonorhinus* sp.

1?; **Fi-19E**; Perinet, 29 May1991 (foresta pluviale).

**Remarks**: Specimens of this order were previously known from 18 humid forest sites on Madagascar; none of the species has been named (Wesener 2014b).

**Order Chordeumatida**

*Betscheuma* spp.

1 M; **Fi-04C**; 5 km S. di Ambalamanakana (strada Ambositra-Fianarantsoa), in foresta, coll. 10 May1991. 1 F; **Fi-24D**; RNI Andohahela, pI, versante E, ca. 300 m, lettiera vagliata, 24-26 May1991. 1 M, 1 F; **Fi-14A**; Mahavelona (=Foulpointe), N. die Tamatave, foresta litorale, lettiera vagliata, 31 May1991. 2 M; **Fi-16B**; Mahavelona (= Foulpointe), N. die Tamatave, foresta litorale, lettiera vagliata, 31May1991. 4 M; **Fi-32B**; Perinet, 1000 m, 8 Oct1989. 2 larvae; **Fi-28D**; Is. Sainte Marie, foreste di Kalalao, 3 Oct1989. 3 larvae; **Fi-xx**; Manjakaptompo, 2000 m, 6 Oct1989.

**Remarks**: Representatives of the Chordeumatida, a group absent from sub-Saharan Africa, were first recorded from Madagascar in the 1990s (Mauriès 1994; 1998). Currently, only species of the genus *Betscheuma* Mauriès, 1994 is known from the island. The endemic genus *Betscheuma* Mauriès, 1994 is closely related to Indian taxa (Enghoff et al. 2015).

**Order Polydesmida**

**Remarks**: Numerous specimens are females or larvae and could not be determined; therefore, only species which could be determined at least to genus are listed.

Family Dalodesmidae

*Dalodesmus* spp.

1 F; **Fi-30A**; Mt d'Ambre 900 m, c/o grande cascade, 26 Sept1989. 1 M; **Fi-24E**; RNI Andohahela, pI, versante E, ca. 300 m, lettiera vagliata, 24-26 May1991. 1 M, 1 F; **Fi-zz**; Grotta di Anjohibe, 12 Sept1989. 1 M, 1 F; **Fi-zz**; Grotta di Anjohibe, 12 Sept1989. 5 juveniles; **Fi-37B**; Montagne d'Andrangoatra (a N. diSambava), 29 Sept1989.

**Remarks**: *Dalodesmus* Cook, 1896 species are the only Polydesmida (except for *Phymatodesmus*) which are indigenous to the island (Enghoff 2003). The remaining Polydesmida fauna constitutes introduced taxa.

Family Paradoxosomatidae

*Oxidus gracilis* (Koch, 1847)

> 30?; **Fi-33A**; Ranomafana, NE Fianarantsoa, foresta, 11May1991. 3 immatures; **Fi-35C**; Ranomafana, sotto cortecce di alteri morti, giardini, 12 May1991. > 5?; **Fi-12B**; Nosy Be, foresta di Lokobe, 16 Sept1989. 5 ♂ & F; **Fi-17C**; Nosy Be, spioggia Ambatoloaka, 15 Sept1989. 5?; **Fi-18A**; Valle del Sambirano, 10 km SE Ambanja, 21Sept1989. 30?; **Fi-32A**; Perinet, 1000 m, 8 Oct1989. 4?; **Fi-20B**; Manjakatompo, c/o station Pisciculture, 1700 m, 5 Oct1989. >5; **Fi-x1**; Antananarivo, Parco Tsimbazaza, 7 Sept 1989. 1 M; **Fi-27B**; Nosy Komba, spiaggi e dint. 17.Sept 1989. 6?; **Fi-x2**; Antsirabe, in giardini di citta, 9 May 1991.

*Orthomorpha coarcata* (de Saussure, 1860)

5 F; **Fi-29B**; Mt. d'Ambre 1100 m, 25 Sept 1989.

*Chondromorpha xanthotricha* (Attems, 1898) **new record for Madagascar**

1 F; **Fi-32F**; Perinet, 1000 m, 8 Oct 1989.

**Remarks**: All three paradoxosomatids are common tropical tramp species (Shelley and Lehtinen 1998, Likhitrakarn et al. 2017).

**Order Spirobolida**

Family Spirobolellidae

*Hylekobolus andasibensis* Wesener, 2009

4 immatures; **Fi-19D**; Perinet, 29 May 1991 (foresta pluviale).

*Hylekobolus* spp.

3 F, 1 immature; **Fi-05E**; PN Ranomafana, foresta, 11 May 1991.

Family Pseudospirobolellidae

*Pseudospirobolellus avernus* (Butler, 1872) **new record for Madagascar**

1 M; **Fi-35E**; Ranomafana, sotto cortecce di alteri morti, giardini, 12 May 1991.

**Remarks**: Tropical tramp, also known from the Comoros (VandenSpiegel and Golovatch 2007) and the Seychelles (Golovatch and Korsós 1992).

Family Pachybolidae

*Aphistogoniulus infernalis* Wesener, 2009

1 F, 1 immature; **Fi-06A**; Andohahela pI, versante E, NW Ft. Dauphin, ca. 300 m, foresta pluviale, lettiera vagliata, 24-26 May 1991.

**Remarks**: This locality fits very well in the known distribution of the species (Wesener et al. 2009b, Wesener et al. 2011). This species has been classified as "endangered" in the IUCN Red List (Rudolf and Wesener 2017a).

*Ostinobolus rufus* Wesener, 2009

1 F; **Fi-07B**; Andohahela pI, versante E, NW Ft. Dauphin, ca. 300 m, foresta pluviale, lettiera vagliata, 24-26 May 1991. 1 F, 1 immature, **Fi-11B**; Andohahela, p1, 500-600 m, foresta lettiera vagliata, 25 May1991; 1 immature M; **Fi-24G**; RNI Andohahela, p1, versante E, ca. 300 m, lettiera vagliata, 24-26 May1991.

**Remarks**: This species is widespread in SE Madagascar, apparently being present in every humid forest that was sampled (Wesener et al. 2009b). This species is classified as "near threatened" in the IUCN Red List (Rudolf and Wesener 2017b).

*Ostinobolus subterraneus* Wesener, 2009

1 F; **Fi-09D**; SE Tolagnaro, dint. Spiaggia Libanona, 23 May 1991.

**Remarks**: This species, only known from lowland forests surrounding Fort Dauphin (Wesener et al. 2009) is currently classified as "critically endangered" in the IUCN Red List (Rudolf and Wesener 2017c). The presence of this species in a habitat slightly modified by humans (although almost 30 years ago) is an indication of a higher resilience than expected of this species to forest removal and human disturbance.

Granitobolus cf. andohahelensis Wesener, 2009

1 M, 4?; **Fi-11A**; Andohahela, p1, 500-600 m, foresta lettiera vagliata, 25 May 1991.

**Remarks**: This species has already been recorded from the area, albeit at higher elevations (Wesener et al. 2009a). The species is listed as "near threatened" in the IUCN Red List (Rudolf and Wesener 2017d).

*Granitobolus* spp.

1 F; **Fi-07E**; Andohahela pI, versante E, NW Ft. Dauphin, ca. 300 m, foresta pluviale, lettiera vagliata, 24-26 May 1991. 1 immature male; **Fi-22A**; dint Evatra, 25 km NE Fort Dauphin, foresta litorale, 23 May 1991.

*Riotintobolus* spp.

1 F; **Fi-09B**; SE Tolagnaro, dint. Spiaggia Libanona, 23 May 1991. 1 M, 1 immature; **Fi-10A**; Andohahela, 6-12Jun-Dec 1991, leg B. Randriamampionona.

*Trigoniulus corallinus* (Gervais, 1847)

1 F; **Fi-17B**; Nosy Be, spioggia Ambatoloaka, 15 Sept 1989.

**Remarks**: Widespread tropical tramp (Shelley and Lehtinen 1999).

*Dactylobolus bivirgatus* (Karsch, 1881)

1 F; **Fi-17A**; Nosy Be, spioggia Ambatoloaka, 15 Sept 1989. MK & F; **Fi-x3**; Sambava, 29 Sept 1989.

**Remarks**: The only indigenous Malagasy Spirobolida that is not a strict endemic to Madagascar. Also occurs in the Comoros (Rollard and Golovatch 2012) and Seychelles (Golovatch and Korsós 1992). On Madagascar only known from humid forests in coastal areas in the northern half of the island.

**Order Spirostreptida**

**Remarks**: Numerous specimens are females or larvae and could not be determined; therefore, only species which could be determined are listed.

Suborder Cambalidea

*Glyphiulus granulatus* (Gervais, 1847) **new record**

2 M; **Fi-25A**; Nosy Be, c/o Cascata, 18 Sept 1989.

**Remarks**: This is a tropical tramp species, already recorded from the Comoros (VandenSpiegel and Golovatch 2007) and Seychelles Islands (Golovatch and Korsós 1992).

Cambalidea indet. cf. Iulomorphinae.

1 M, 5 F; **Fi-33B**; Ranomafana, foresta, 11 May 1991. 1 M, 5?; **Fi-34A**; Vohiparara, 13 km W. die Ranomafana, foresta secondaria, 10 May 1991. 2 M, 11 F; **Fi-07F**; Andohahela pI, versante E, NW Ft. Dauphin, ca. 300 m, foresta pluviale, lettiera vagliata, 24-26 May 1991. 1 M, 5?; **Fi-09C**; SE Tolagnaro, dint. Spiaggia Libanona, 23 May .1991. 12?; **Fi-11A**; Andohahela, pI, 500-600 m, foresta lettiera vagliata, 25 May 1991. 9?; **Fi-24C**; RNI Andohahela, pI, versante E, ca. 300 m, lettiera vagliata, 24-26 May 1991. 2 M; **Fi-14A**; Mahavelona (=Foulpointe), N. die Tamatave, foresta litorale, lettiera vagliata, 31May 1991. 8?; **Fi-13B**; Tampolo, 10 km N. Fenerive, foresta, lettiera, 1Jun 1991. 1?; **Fi-22A**; dint Evatra, 25 km NE Fort Dauphin, foresta litorale, 23 May 1991. 2?; **Fi-36B**; S. fra Ampanihy e Beloha, ca. 20 km d. Beloha, boscaglia, 22 May 1991.

**Remarks**: Undetermined Iulomorphine specimens, already mentioned previously (Enghoff 2003), seem to be widespread on Madagascar. The occurrence in disturbed habitats, often alongside introduced tropical tramp species, suggest that they may belong to one (or more?) introduced species.

Suborder Spirostreptidea, family Spirostreptidae

*Eumekius antimena* (de Saussure & Zehntner, 1901)

1 M; **Fi-12A**; Nosy Be, foresta di Lokobe, 16.ix.1989. 3 M; **Fi-27A**; Nosy Komba, spiaggi e dint. 17 Sept 1989.

## Discussion

### Relationships and biogeography of the newly described species

*Zoosphaerium mangabe* sp. nov. shows an unusual distributed pattern, linking lowland rainforest of the island Nosy Mangabe to the nearby mountain forest of Marojejy. The close link (9.9% p-distance in the COI) of *Z. mangabe* sp. nov. to the morphologically very different (Sagorny and Wesener 2017) *Z. minutus* from northern Madagascar is surprising.

*Zoosphaerium bartolozzii* sp. nov. seems most closely related to *Z. tigrioculatum* based on morphological characters such as the presence of three sclerotized crenulated teeth on podomere 3 of the anterior telopod. Both species were collected from the humid evergreen forests present in the south-east of Madagascar, at specific small microclimatic refugees. *Zoosphaerium taitii* sp. nov. seems more similar to *Z. isalo* due to the absence of sclerotized crenulated teeth on podomere 3 of the anterior telopod (Wesener 2009, Wesener et al. 2010a). *Zoosphaerium taitii* sp. nov. was recorded from the Zombitse forest and *Z. isalo* from the Isalo National Park which both lie in the south-west of Madagascar. *Zoosphaerium taitii* sp. nov. occurs in sympatry with *Z. album*, a species belonging to a different species group (Wesener 2016).

### Notes on introduced species

Among the Diplopoda collection of the museum "La Specola" from Madagascar, 30% of the specimens represent introduced species. They belong to four orders: Polyzoniida, Polydesmida, Spirobolida, and Spirostreptida.

Order Polyzoniida: *Rhinotus purpureus* is a worldwide introduced species (see Peck and Shear 2000, Shelley and Golovatch 2011), native to Central America and Caribbean islands (Golovatch and Korsós 1992). In Madagascar, they have actively conquered the majority of the vegetated land areas except dry ecosystems (Wesener 2014a).

Order Polydesmida: So far eleven species have been recorded from Madagascar, of which only the seven members of the genus *Dalodesmus* and the single species of *Phymatodesmus* are indigenous, while four are introduced species (Enghoff 2003). Among the collections of the Museum “La Specola” there were three of the introduced species, of which two are common tramps *Oxidus gracilis* and *Orthomorpha coarcata* (see Stoev 2004, Kime and Enghoff 2012, Rollard and Golovatch 2012, Jovanović et al. 2016, Nguyen et al. 2017). The third species, *Chondromorpha xanthotricha* is also a common tropical tramp (Likhitrakarn et al. 2017), but this is the first record for Madagascar.

Order Spirobolida: Madagascar hosts the highest diversity of Spirobolida in the world, with a good degree of endemism (15 endemic genera, Wesener et al. 2009, Wesener 2011). Aside from the previously recorded *Trigoniulus corallinus*, a widespread tramp (see Shelley 1998, Korsós 2004, Shelley et al. 2006), one other common Spirobolida tramp species is recorded for the first time from Madagascar, *Pseudospirobolellus avernus*, as is the spirostreptid *Glyphiulus granulatus*.

### Impact of introduced species

The seven tropical tramp species found in the collections of the Museum “La Specola” have been introduced on this island by human activity. Millipedes are often introduced along with soil or plants (Decker and Tertilt 2012). The previously known tramp species are widespread on Madagascar (Enghoff 2003, Wesener 2014a) and along with the three new records, they account for > 25% of the millipede collection of the Museum “La Specola”, which clearly demonstrates human influence on this island. These seven tramp species are recorded worldwide as introduced species. Studies have suggested that introduced species may have chances to replace the indigenous species existing in that region (Shelley and Golovatch 2011, Wesener 2014a). In addition to the continuous deforestation, the widespread presence of these introduced millipede species could be an understudied but severe threat to the endemic and unique millipede fauna of Madagascar.

## Supplementary Material

XML Treatment for Zoosphaerium

XML Treatment for Zoosphaerium mangabe

XML Treatment for Zoosphaerium bartolozzii

XML Treatment for Zoosphaerium taitii
